# All Happy Emotions Are Alike but Every Unhappy Emotion Is Unhappy in Its Own Way: A Network Perspective to Academic Emotions

**DOI:** 10.3389/fpsyg.2020.00742

**Published:** 2020-04-30

**Authors:** Markus Mattsson, Telle Hailikari, Anna Parpala

**Affiliations:** Centre for University Teaching and Learning (HYPE), University of Helsinki, Helsinki, Finland

**Keywords:** network model, factor model, academic emotions, emotion research, reflective model

## Abstract

Quantitative research into the nature of academic emotions has thus far been dominated by factor analyses of questionnaire data. Recently, psychometric network analysis has arisen as an alternative method of conceptualizing the composition of psychological phenomena such as emotions: while factor models view emotions as underlying causes of affects, cognitions and behavior, in network models psychological phenomena are viewed as arising from the interactions of their component parts. We argue that the network perspective is of interest to studies of academic emotions due to its compatibility with the theoretical assumptions of the control value theory of academic emotions. In this contribution we assess the structure of a Finnish questionnaire of academic emotions using both network analysis and exploratory factor analysis on cross-sectional data obtained during a single course. The global correlational structure of the network, investigated using the spinglass community detection analysis, differed from the results of the factor analysis mainly in that positive emotions were grouped in one community but loaded on different factors. Local associations between pairs of variables in the network model may arise due to different reasons, such as variable A causing variation in variable B or vice versa, or due to a latent variable affecting both. We view the relationship between feelings of self-efficacy and the other emotions as causal hypotheses, and argue that strengthening the students’ self-efficacy may have a beneficial effect on the rest of the emotions they experienced on the course. Other local associations in the network model are argued to arise due to unmodeled latent variables. Future psychometric studies may benefit from combining network models and factor models in researching the structure of academic emotions.

## Introduction

The emotional experiences of students have been a topic of intensive research efforts during the previous two decades. The term “academic emotions” was introduced to refer to emotions that arise in different academic settings and that are directly linked to academic learning, instruction and achievement (Pekrun et al., 2002). Research in this area is important, as academic settings are permeated by emotions ranging from test anxiety (Zeidner, 1998) to flow experiences (Pekrun, 2006). The emotions that students and teachers experience in academic settings have direct effects on how they process information, where they direct their attention and what they remember (Pekrun et al., 2002). Further, the emotional state of students is an important predictor of how they perform academically: just to give one example, test anxiety is associated with worse learning outcomes (Pekrun et al., 2002; Ketonen and Lonka, 2012), while hopefulness and confidence are related to better outcomes (Asikainen et al., 2017). Emotions determine to a large extent whether students want to be a part of the academic community, to engage with it (Pekrun and Linnenbrink-Garcia, 2012). Understanding the emotions that students experience in academic settings is, then, important both in its own right and because of the practical consequences of various academic emotions.

Academic emotions are often studied using self-report instruments such as the Academic Emotions Questionnaire (AEQ; Pekrun et al., 2011), Academic Emotions Scale (AES; Govaerts and Grégoire, 2008) and the Student Experience of Emotions Inventory (SEEI; Trigwell et al., 2012). The three instruments are related in that the AEQ was first developed based on the control value theory of achievement emotions (Pekrun et al., 2007), the AES was then constructed based on the AEQ, and the SEEI used the AES as inspiration. A brief summary of the theoretical background is, then, in order.

In Pekrun’s theory (Pekrun et al., 2007), *achievement emotions* refer to emotions related to academic activities or the outcomes of such activities. The term *control* refers to individuals’ appraisals of whether they are in control of the achievement activities and *value* to appraisals of the subjective value that such activities have for the individuals. A three-dimensional taxonomy of the emotions is formed by referring to the emotions’ (1) *object focus* (emotions related to achievements vs. their outcomes), (2) *valence* (positive vs. negative emotions) and (3) *activation* (activating vs. deactivating emotions). In addition, the building blocks of emotions, such as anxiety, include affect (e.g., feeling tense), cognition (worrying), motivation (impulse to escape) and physiological responses (shaky hands, pounding heart).

**FIGURE 1:**
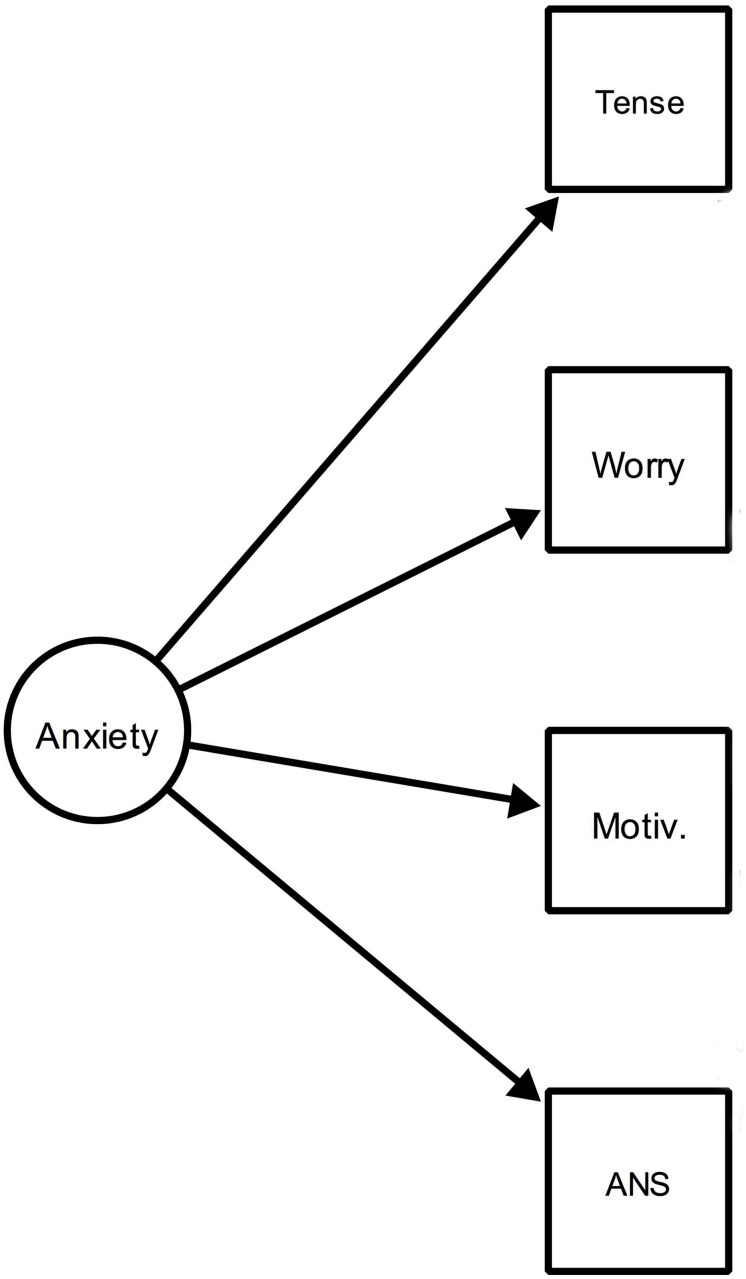
Schematic representation of a reflective model of anxiety. Tense, feeling tense; Worry, worrying about what will happen; Motiv, having the motivation to escape from the situation; ANS, activation of the autonomic nervous system.

The AEQ includes items related to each of the four building blocks of the emotions, while in the AES and the SEEI, items related to cognition and motivation were dropped. The relationship between the items and the factors is formalized using factor models, also known as reflective measurement models (Bollen and Bauldry, 2011); see e.g., Figure 1 in Pekrun et al. (2011). Figure 1 below shows a schematic representation of such a model for the four building blocks of anxiety assumed in the control value theory (please note that Figure 1 serves to demonstrate the general idea of reflective measurement models and is based on no data).

An important question related to interpreting such models concerns the nature of the relationship between the emotion (ellipse) and its indicators (squares). In short, the two main alternatives appear to be (1) *realism*, according to which the phenomenon of interest, in this case the emotion of anxiety, exists independently of measuring it and causes variation in the four component psychological processes and (2) *constructivism*, which views the latent variables as (social) constructions whose definitions can be agreed upon by researchers and that do not necessarily exist independently of measuring them (Borsboom et al., 2003).

We concur with Borsboom et al. (2003) that a coherent interpretation of reflective models entails a realist ontology, that is to say, in this case, the assumption that the emotions exist independently of measuring them, that variation in the emotion precedes variation in the indicators, and that it is sensible to ask which factor model is the correct representation of the emotions instead of asking merely which one of them is empirically adequate or offers the best fit to data (for a more thorough discussion of these points, see Borsboom et al., 2003, pp. 208–210). Previous research on academic emotions has had an essentially realist flavor, building on reflective latent variable models that assume the existence of either several discrete emotions, two dimensions of valence (positive/negative) and activation (activating/deactivating) emotions or a combination of these (Govaerts and Grégoire, 2008; Pekrun et al., 2011; Trigwell et al., 2012). However, the issue has not been explicitly discussed in previous research on academic emotions.

However, it can be questioned whether latent variable models are, in fact, the most appropriate representation for academic emotions. This is because interactions among the components of the emotions are emphasized in the control value theory and the component process model (Scherer, 1984, 2009) that inspired the theory. This thinking is exemplified in statements such as “…we view emotions as involving sets of interrelated psychological processes. Affective, cognitive, physiological, and motivational component processes may be the most important” (Pekrun et al., 2002, p. 95) and “students experience a rich and intense emotional life in academic settings, suggesting that reductionist conceptions of students’ emotions may fall short of adequately covering this domain” (Pekrun et al., 2002, p. 94). Such interactions among components are explicitly modeled as a dynamic system by Scherer (2009) based on the idea of emotions as emergent processes; this idea is obviously in line with the anti-reductionist spirit of Pekrun et al. (2002). A further property of latent variable models that appears to be incompatible with the component process model is that manipulating the observed variables (i.e., making one feel more tense) should have no effect on the latent variable (anxiety), as in reflective measurement models the former are assumed to be causally passive indicators of the latter (Edwards and Bagozzi, 2000).

Importantly, according to the component process model, emotions are best modeled using dynamic systems models, which embody different sorts of feedback and feedforward mechanisms between the subsystems underlying the emotions (Scherer, 2009). Because of this, “there is no simple, unidirectional sense of causality” in the model (Scherer, 2009). Interestingly, a novel psychometric approach that is compatible with the idea of emotions as emergent phenomena arising from complex interactions among their component parts has been introduced in the recent years. This approach is known as *network psychometrics* and it has been used to model phenomena ranging from psychopathology (Borsboom, 2017) to personality (Cramer et al., 2012), driver behavior (Mattsson, 2019), and students’ engagement to their studies (Korhonen et al., 2019).

**FIGURE 2:**
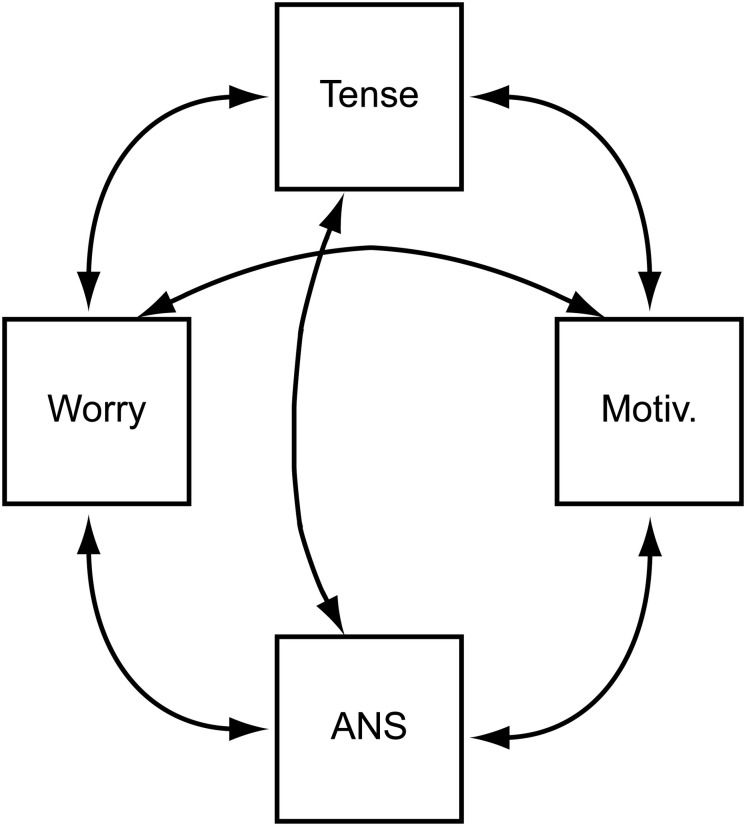
A schematic network model for the emotion of anxiety.

A psychometric network model (Epskamp et al., 2018) consists of direct relationships between the observed variables that remain when controlling for the effects of all the other variables in the network. In these models, latent variables are best understood as emergent properties produced by local interactions among the observed variables. For example, the emotion of test anxiety (the latent variable) would arise when anxious thoughts (“I am going to fail the exam”) interact with the affective state characteristic of anxiety, the bodily feeling of not getting enough air and the motive to withdraw from the situation. In a network model, the relationship of the observed variables to the emergent latent variable is that of parts to a whole, i.e., mereological composition (Cramer et al., 2012; Markus, 2014) rather than one of measurement (Borsboom and Cramer, 2013). Still, network models can accommodate the equivalent of a latent variable in a *community* of nodes: a group of nodes that have strong pairwise links with one another, and only weak links to outside the community. Another way of understanding communities is to consider randomly chosen pairs of nodes. If they are part of the same community, they are more likely to share a strong link than if they belong to different communities. Finally, the network models build on the assumption that the nodes of the network are unique components in the sense of having causal relationships with the rest of the network that are unique to the node in question (Cramer et al., 2012). A network model of emotions as emergent properties of the interaction among their component parts is shown schematically in Figure 2 (please note that Figure 2 serves to demonstrate the general idea of network models and is based on no data).

The edges (connections) among the nodes (variables) in a network model may signify different things. An edge between nodes A and B may occur because A is the cause of B or vice versa, even though edges may also reflect the influence of a latent variable not included in the network that influences both. Promising new methods for investigating whether a connection reflects a pairwise association or the presence of an unmodeled latent variable have been developed, but they are beyond the scope of the present contribution (van Bork et al., 2019). In addition, an edge may indicate a logical relationship of entailment between the two variables; for instance, if a self-report instrument contains questions related to being able to walk 100 m and being able to walk 1 km, an affirmative response to the latter logically entails an affirmative answer to the former (Kossakowski et al., 2016). Finally, a connection may exist just because the things signified by the variables commonly co-occur in a society such as ours.

Further, psychometric network models are a natural tool for representing phenomena that develop in time and which are fundamental in the component process model of emotions (Scherer, 2009). Consider, for instance, the relationship between frustration and anger: a steady increase in frustration may first result in a steady increase in anger, but when a threshold is reached, the person’s anger may abruptly step up to a much higher level, resulting in the person suddenly flaming up in anger. In network theory, the term *hysteresis* is used when referring to a temporary change in one factor causing a permanent or long-lasting change in another factor (Borsboom, 2017); in the example of Scherer (2009), the person becoming angry may remain so after the factor causing the frustration has been removed. Even though we do not discuss such dynamics in this contribution, the fact that psychometric network models can naturally account for them motivates their use in modeling academic emotions.

In summary: The control value theory of achievement emotions has served as the theoretical backdrop for the AEQ, and the AES and SEEI have been derived from the AEQ and the theory underlying it. The control value theory, for its part, has been developed based on (among other things) the component process model of emotions, which views emotions as arising through interaction of their component parts (such as physiological activation, cognitions, feelings, and motivations). Because of this, we have argued that the recently developed network approach to psychometrics serves as an appropriate framework for analyzing psychometric data on academic emotions.

When introducing a new self-report instrument, or translating an existing one into a new language, validating it is commonly seen as an essential part of the research process. In the studies that originally reported the three questionnaires (AEQ, AES, and SEEI), the question was approached from the point of view of *construct validity* (Cronbach and Meehl, 1955; Messick, 1987; Kane, 2006). Construct validity, as originally presented by Cronbach and Meehl (1955) investigates *nomological networks* of the constructs mentioned in a theory; in other words, what construct validity requires is the explication of scientific laws connecting the central constructs (and their operationalizations). In the context of academic emotions, this would mean forming scientific laws concerning, say, emotions and academic performance and the relationships of theoretical terms (such as social phobia) and observable properties (such as sweaty palms). In the human sciences, explicating such lawlike relationships has proven to be a tall order (Borsboom et al., 2009), and current analyses of construct validity concentrate on correlational relationships instead of scientific laws. Typical analyses concentrate on the internal structure of constructs (factor analyses concerning convergent and divergent validity) and on correlating the constructs with various other constructs that are deemed relevant. A typical analysis workflow in a construct validation study in the educational sciences is described by Knekta et al. (2019). Latent variable models are used for investigating the internal structure of the instrument and variables are dropped to create clear subgroups of intercorrelated variables. This process is then iterated a suitable number of times, with suitability defined using different heuristic quantitative indices and the researchers’ judgment. Then, the resulting test scores are correlated with external variables. The AEQ, AES, and SEEI were developed essentially in this manner. For instance, developing the AES and SEEI involved fitting a series of latent variable models and the resulting SEEI scores were then correlated with approaches to learning variables.

In this contribution, we examine the internal structure of a Finnish self-report instrument for assessing academic emotions. We present a latent factor model and a network model of the instrument, and compare the kinds of validity-related inferences that one can draw based on the two analyses. We highlight the different practical decisions that are likely to result from viewing academic emotions as latent variables causing variation in their indicators vs. emergent phenomena that arise through the interactions of their parts.

It is noteworthy that the choice between latent factor models vs. network models implies notably different goals for validating the instrument. While factor analysis aims at identifying maximally intercorrelated variables, network models are based on the idea of “network components” (Cramer et al., 2012), i.e., nodes that have unique relationships with the rest of the network. While creating redundant items – for instance by writing the items such that their contents are (nearly) synonymous – is a good way of maximizing their intercorrelations, such practice does not lead to unique network components. Rather, including items with unique contents and that describe phenomena that are likely to be causally interconnected is likely to lead to network components in the current sense. In short, the factor models and network models are likely to lead to quite different practical conclusions regarding the inclusion of individual items. It is of interest that validation based on network models allows keeping theoretically important variables aboard even if they do not correlate strongly with (many) other variables.

Incidentally, forming highly redundant items will also maximize reliability, when reliability refers to the degree of interrelatedness of the items (e.g., Cronbachs alpha). Further, calculating alpha reliability presupposes the unidimensionality and the tau equivalence (equal factor loadings for all the items) of the items rather than providing evidence on the presence or absence of unidimensionality (Kline, 2011). In this sense, it can be considered a rather uninformative statistic: it says, at best, that items that have been created to be highly interrelated actually are so. Reliability indices that apply to network models are under development but have not been published at the writing of the present contribution (Christensen et al., 2019); in the future, such indices are likely to play an important role in analyzing the structure of network models.

Identifying similar groups of variables is something that can be investigated based on both factor analysis and community analysis with network models. In network models, communities are groups of items that are strongly interconnected, while having weaker connections with the rest of the network (Fortunato, 2010; please see the “Materials and Methods” section for a more detailed description of a network community). It can be argued that community analysis is a more straightforward method for reaching this objective, because community analysis creates such groupings of variables automatically, without the researcher needing to specify a given rotation method as in a factor analysis. We highlight such differences between the models in the Discussion.

One further practical benefit of creating network models instead of or in addition to factor models is that they help avoiding straightforward interpretations of why certain items are correlated. Whereas factor analysis encourages naming the common denominator for the items that load on a factor, network analysis captures more subtle relationships between the items. Finally, it is important to note that for every psychometric network model there exists a latent variable model that fits the same data equally well (Molenaar, 2010; Epskamp et al., 2018). Still, the two kinds of models involve very different (causal) assumptions on the nature of the phenomenon under investigation. Because of this, the choice between the models needs to be done on substantive grounds.

This contribution reports a Finnish version of the AEQ that is derived from the AEQ, AES, and the SEEI. The study reports factor analyses and network analyses of the questionnaire and compares the kinds of validity-related conclusions that can be drawn based on the two methods. We conclude by suggesting ways to further develop measurement instruments related to academic emotions and by considering the theoretical background assumptions related to network models and latent variable models of academic emotions.

The rest of the manuscript is organized as follows. First, we introduce certain background concepts related to using network models. Second, we report the results of performing an exploratory factor analysis (EFA) on our data set that was collected using a Finnish AEQ that was previously developed based on the AEQ (Pekrun et al., 2011), AES (Govaerts and Grégoire, 2008), and the SEEI (Trigwell et al., 2012). Third, we analyze our data set using a network model and calculate associated centrality indices for the nodes of the network. A change in a node deemed central in this sense may lead to changes in other nodes at least for some of the participants. Fourth, we compare the ways that the phenomenon of academic emotions becomes conceptualized when analyzed using a network model vs. a factor model; similarly, we assess the kinds of practical conclusions and suggestions that it is possible to draw based on either model. We also interpret our results from the point of view of the various dimensions that can be used in analyzing the phenomenon of emotions. Finally, we conclude by offering certain recommendations and suggestions for further research.

## Materials and Methods

### Participants and Data

The data consisted of Bachelor students studying in the field of technology and engineering. They took a lecture course consisting of lectures, homework, a midpoint exam and a final exam. A total of 350 students enrolled on the course, of which 323 students were active (returning at least one homework assignment). A total of 241 responses were obtained, while for technical reasons three responses were discarded. The resulting dataset is included as Supplementary Data Sheet 1. The students had an incentive to take part in the study as movie tickets were raffled among the respondents and they were awarded an extra homework point.

The course is known to be extremely demanding and many students fail to pass it, forcing numerous students to repeat it. 171 students (72% of participants) took part in the course for the first time, 43 (18%) for the second time and 25 (10%) for the third time or more. 87% of the respondents were male, 13% female, while 49% were first year students and 23% second year students. The rest had begun their studies earlier. The teachers claim that because of the demanding content the students have negative attitudes and emotions toward the course. Therefore, different emotions were expected to arise during the course. The data was obtained using an electronic questionnaire focusing on academic emotions during the course, just before the midpoint exam.

The study was exempt from ethics approval as per the guidelines of Finnish Advisory Board on Research Ethics (National Advisory Board on Research Ethics, 2009). Specifically, (1) written informed consent was inferred from the participants returning the questionnaire forms, while (2) risks related to social harm and confidentiality were minimized by storing the original questionnaire forms in a locked space, and by only using anonymized versions of the data sets in all data analyses.

### Development of the Questionnaire

A questionnaire related to academic emotions was developed by translating and modifying items from several prior questionnaires. First, the Students Experience of Emotions Inventory (SEEI; Trigwell et al., 2012) was taken as a basis for the instrument as it focuses on course level emotions among university students. The SEEI was based on the AES developed by Govaerts and Grégoire (2008), which focused on state academic emotions occurring in a specific context. As the SEEI contains only two items on anxiety, an additional item on homework-related anxiety was included from the AES. In addition, SEEI does not include items related to the experiences of joy and despair, which also appear in an academic context (Pekrun et al., 2002). Therefore, items related to these emotions were added using the items from Achievement Emotions Questionnaire (AEQ; Pekrun et al., 2011) as a basis. The items were first translated into Finnish and modified to suit the course context. In this process items referring to the final test were modified to focus on the midpoint and final exam.

After the first round of modifying the items a focus group interview was conducted. Students participating in the focus group were asked to fill in the questionnaire and were subsequently interviewed. In the interview they were asked to evaluate each item and its content in the context of their studies. If the students felt that the item did not suit the context of their studying the item was re-modified. During the process it became clear that anger was considered a negative emotion of such intensity that students did not report experiencing it during their studies. Therefore, items mentioning *anger* were replaced with items containing milder expressions such as *irritation*. Moreover, *pride* is an academic emotion rarely expressed in the Finnish context, because it carries a strong connotation of self-importance. For this reason mentions of *pride* were replaced with expressions such as *being extremely satisfied with one’s performance*. This modification process was done among the experts in university pedagogy and students studying behavioral sciences bearing in mind the Finnish context. The original items and back-translated versions are presented in Table 1. The back-translations are literal translations from Finnish into English, provided for the convenience of English-speaking readers. Responses to all question items were given using a 5-point Likert scale (1 = Completely disagree, 5 = Completely agree).

**TABLE 1:** Questionnaire items used in the present study.

**Item no.**	**Item in English (translated from Finnish by the lead author)**	**Corresponding item in original**	**Item code in original^ψ^**	**Latent variable in original**
1	I find studying the course contents inspiring	I am motivated to go to this class because it’s exciting*	P11, CJOM1B	Enjoyment
2	I feel optimistic about my preparation for the exam on this course	I feel optimistic about my preparation for the assessment in this course	T9	Positive emotions
3	I am extremely happy with my learning on this course	I feel proud of my progress in this course	T25	Positive emotions
4	Completing the assignments on this course irritates me	I get angry with this course*	T15	Anger-Boredom
5	I am bored by this course	I am bored by this course	T3	Anger-Boredom
6	Studying the materials of this course feels hopeless	I feel hopeless when I think about studying	P95, LHLA1B	Hopelessness
7	I am extremely happy with the way I have done with the homework in this course	I get a feeling of pride as a result of my work on this course	T21	Positive emotions
8	I am fed up with the contents of this course	The content of this course irritates me*	T7	Anger-Boredom
9	I feel like I cannot make it through this course	I start to think that no matter how hard I try I won’t succeed on the test*	P185, THLC3D	Hopelessness
10	I feel ashamed not having prepared better for this course	I feel ashamed not having prepared better for this course	T17	Anxiety-Shame
11	I feel anxious when I am doing the homework assignments on this course	I feel anxious when I am doing exercises for the math exam	G5	Anxiety
12	I feel ashamed thinking I might have not prepared adequately for the exam in this course	I feel ashamed thinking I might have not prepared adequately for the assessment in course	T5	Anxiety-Shame
13	The contents and the assignments on this course make me unhappy	I feel annoyed when trying the learning activities for this course*	T23	Anger-Boredom
14	When I think about this course, I become anxious	When I think about this course, I become panicky	T8	Anxiety-Shame
15	I enjoy learning new things on this course	I enjoy acquiring new knowledge	P139, LJOA3D	Enjoyment
16	I am sure that I will do well on this course	I am sure that everything is going well in this course	T18	Positive emotions
17	I find the course exam a pleasant challenge	For me the test is a challenge that is enjoyable	P204, TJOC3D	Enjoyment
18	The learning activities for this course are not interesting	The learning activities for this course are not interesting	T19	Anger-Boredom
19	I am embarrassed when I cannot complete the homework assignments on this course	I am embarrassed when I cannot contribute to learning activities	T11	Anxiety-Shame
20	Contributing to discussions or asking questions on this course makes me anxious	Contributing to discussions in class makes me anxious	T29	Anxiety-Shame
21	If I ask a question in class that others already certainly know the answer to, I feel embarrassed	If I ask a question in class that others already understand, I feel embarrassed	T30	Anxiety-Shame
22	Mistakes I make on the course (e.g., during the mathematical excercises) or when doing homework embarrass me	When I get an idea wrong in class I feel embarrassed	T24	Anxiety-Shame
23	I have lost hope of doing well in the course exam	I have lost all hope that I have the ability to do well on the exam	P164, THLC1B	Hopelessness
24	In the beginning of this course I was confident that I will do well on this course	I generally feel confident that I will do well when I begin a course	T22	Positive emotions
25	I am extremely happy with the way I have invested my efforts in studying on this course	I am proud of the way I am contributing to this course	T16	Positive emotions

*Our item has no directly equivalent item in the original questionnaire; this is the closest equivalent. ^ψ^Item codes: T, item from Trigwell et al. (2012); P, item from Pekrun et al. (2005); G, item from Govaerts and Grégoire (2008). Numbers are the item numbers from the original, the sequence of letters and numbers additional codes from the original.

### Analysis

Means, standard deviations and the polychoric correlations among the emotion variables are reported in Supplementary Table S1. Polychoric correlations are a method for quantifying linear dependencies among ordinal variables. Calculating polychoric correlations entails the assumption that there exists an underlying normally distributed variable that has been “cut” into the ordinal categories; polychoric correlations function as an estimate of these assumedly normally distributed variables.

#### Factor Analysis

An exploratory factor analysis was performed using the psych package (Revelle, 2014) in R (R Core Team, 2016) to demonstrate modeling emotions based on a reflective measurement model. The matrix of polychoric correlations was used as an input to minimum residual (minres) factor analysis (Harman and Jones, 1966), followed by oblique rotation using the geomin method. The minres extraction method was used as it has been shown to be superior to the maximum likelihood method when weak factors are present (Briggs and MacCallum, 2003). Geomin rotation was used because simulation studies have shown that it is likely to converge on a simple structure if one is present (Schmitt and Sass, 2011). The suitability of the data to factor analysis was assessed using Bartlett’s test and the Kaiser-Meyer-Olkin test of sampling adequacy. A parallel analysis based on the factor analysis method was performed to assess the number of factors to extract.

#### Network Analysis

All network analyses were performed using the qgraph package (Epskamp et al., 2012) in R (R Core Team, 2016). The networks represent partial polychoric correlations among the variables, with the connection weights determined using the graphical lasso method as implemented in qgraph. The graphical lasso is a regularization method, i.e., an estimation method that involves trading a small amount of increase in bias to a decrease in the variance of the estimates. The graphical lasso does this by applying a penalty to the absolute values of the elements of the inverse covariance matrix, constraining small values to zeroes and all other values slightly closer to zero (Friedman et al., 2008). This is useful as the procedure implements model selection and parameter estimation simultaneously, thus avoiding, for instance, the necessity of calculating *p*-values and correcting them for multiple comparisons. Other benefits of using the lasso include avoiding overfitting models to the sample of data, and an increase in interpretability that results from constraining small values of partial correlations to zero. For more details, see Epskamp and Fried (2016).

In the network graphs, the nodes (the circles) correspond to individual questionnaire items. Connections between the nodes are referred to as “edges.” The width and saturation (the fullness of the color) of the edges among the nodes illustrate the strengths of the lasso-estimated partial polychoric correlations. Edges drawn in green color represent positive associations, while those drawn in dashed red lines represent negative associations.

Positioning the nodes of the network on a two-dimensional surface in a way that optimally reflects their interrelationships is not a trivial problem. In qgraph, this problem is solved by applying the Fruchterman–Reingold algorithm (Fruchterman and Reingold, 1991), which places strongly connected nodes close to each other. Further, nodes that have the overall strongest connections in the network are positioned centrally in the graph, while nodes with weaker connections appear closer to the periphery of the graph (Epskamp et al., 2012).

The resulting networks can be described using the global and local indices that are commonly used in the description of network graphs, such as measures of distance, centrality and clustering. In this contribution, we focus on measures of centrality (Opsahl et al., 2010), which are especially relevant when comparing network models with factor models: while the interchangeability of the indicators is central to reflective (factor) models, in network models the nodes vary in how important or central they are.

##### Centrality measures

Three measures that are commonly used in characterizing the properties of a network graph are briefly summarized below. These indices are known as *strength centrality*, *closeness centrality* and *betweenness centrality.* Strength centrality describes the absolute values of the weights of the connections to a focal node. When a node is strongly connected to its neighborhood, a change in the value of the node will have a large effect on the nodes in its neighborhood. Closeness and betweenness centrality can be understood after defining the distance between a pair of nodes, which in a weighted network is the inverse of the absolute value of their connection weight. Metaphorically, the idea can be described by thinking of the two nodes as cities and the weight of the connection as the size of the road connecting them: if the cities are connected by a highway (a large weight), they are close to one another (in travel time) and vice versa if they are connected by a rural road (a small weight). The closeness centrality of a focal node is defined as the inverse of the sum of its distances to other nodes in the network. A node with a high closeness centrality is easily affected by changes in the values of the other nodes in the network. The betweenness centrality of a focal node is defined as the number of shortest paths between two other nodes that pass through that node. A node with a high betweenness centrality is important for the smooth flow of information through the network: if a node with a high betweenness centrality value were removed from the network, the distances between other nodes would increase considerably. It has been argued that between-subjects network models can be interpreted as aggregations of potentially different within-subject network models (Cramer et al., 2012). Thus, centrality indices need to be interpreted carefully, as it is not clear to which extent they apply as descriptions of individuals. More in-depth information on their properties and examples of the use of centrality indices can be found, e.g., in Opsahl et al. (2010) and Costantini et al. (2015).

##### Network community structure detection

Network community detection is a process of determining groups of nodes that are densely interconnected and have only relatively few connections to nodes outside the group (Fortunato, 2010). Many different community detection algorithms have been developed for different types of networks, such as weighted, directed and signed ones. *Weighted networks* refers to networks where edges have a weight that corresponds to a property of interest. In the network models reported in the current contribution edge weights correspond to lasso-estimated partial correlations. A network is directed when a connection between two nodes runs in one direction only; the network models reported in the current contribution are undirected as partial correlations involve no direction of the relationship. A signed network involves edges that may have either positive or negative values. The presently reported network models are signed, as partial correlations may be either positive or negative.

In this study, we used the spinglass algorithm (Reichardt and Bornholdt, 2006), which is suitable for weighted and signed networks such as the present one. The spinglass algorithm borrows from ideas of statistical mechanics and describes a system of spins that can be in *q* different states. The algorithm likens the nodes of a network with spin variables and examines interactions between neighboring variables with the aim of minimizing the energy (Hamiltonian) of the system; the resulting communities correspond to the spin states (Fortunato and Castellano, 2007). The algorithm rewards edges between variables in the same community and non-edges between those in different communities while penalizing non-edges within communities and edges between communities (Fortunato and Hric, 2016). The minimization of energy is achieved using simulated annealing (Kirkpatrick et al., 1983), which for its part is a heuristic algorithm for efficiently solving complex optimization problems for which exact solutions do not exist.

The spinglass algorithm has been shown to perform well with quite high values of the mixing parameter, which describes the proportion of connections from a focal node to other nodes outside its own community (Yang et al., 2016). It is conceivable that in the present application this proportion quite high, since there are likely numerous reasons for the questionnaire items to correlate. Further, when the network comprises small number of nodes, the spinglass algorithm is known to reliably detect the correct number of communities (Yang et al., 2016).

The algorithm was run using the default values in igraph (Csardi and Nepusz, 2006) in R (R Core Team, 2016). Due to the stochastic nature of the algorithm, 1000 repetitions of the analysis were run, and the most frequently obtained community structure reported.

##### Accuracy and stability of the results

We investigated the robustness of our findings by calculating indices of accuracy and stability of the results. Accuracy refers to how prone the results are to sampling variation, while stability refers to whether the results remain similar with a smaller number of observations (Epskamp et al., 2017). The accuracy of edge weight estimates was assessed by calculating 95% confidence intervals using the non-parametric bootstrap procedure in the R package bootnet (Epskamp et al., 2017). 2500 bootstrap draws were performed. The narrower the confidence intervals are, the more interpretable the edge weight estimates and their differences (Epskamp et al., 2017). The stability of the centrality indices was assessed using the correlation stability coefficient (CS-coefficient), which is defined as the maximum proportion of cases that can be dropped such that with 95% probability the correlation between the original centrality indices and those based on subsets is 0.7 or higher (Epskamp et al., 2017). The minimum recommended value for the coefficient that enables the interpretation of the centrality coefficients is 0.25, while its value should preferably exceed 0.5 (Epskamp et al., 2017). Again, 2500 bootstrap draws were performed.

## Results

### Missing Data

The maximum number of missing values in any single questionnaire item was two, and it appeared that the missing values were inadvertent omissions of questionnaire items. Because of this, it was assumed that the missing data mechanism was *missing completely at random* (MCAR, Schafer and Graham, 2002) and the missing values were dealt with by calculating the polychoric correlation matrices with pairwise deletion of missing values.

### Factor Analysis

Parallel analysis of the emotion items favored a four-factor solution. Accordingly, four factors were extracted and rotated using the geomin oblique rotation. Tentative interpretations of the factors are given in the column headings of Table 2. The complexity scores indicate the presence of some cross-loadings among the items. The communality values and the related uniqueness values are shown in the remaining two columns of Table 2.

**TABLE 2:** Exploratory factor analysis of the emotion items.

**Item**	**Item no.**	**Factor**				***h*^2^**	***u*^2^**	**com**
		**Anxiety and hopelessness**	**Boredom and anger**	**Effort-related em.**	**Negative social em.**			
I feel like I cannot make it through this course	9	**0.77**	–0.01	–0.04	0.02	0.62	0.38	1.0
I have lost hope of doing well in the course exam	23	**0.71**	0.00	–0.19	0.17	0.73	0.27	1.3
I feel anxious when I am doing the homework assignments on this course	11	**0.71**	**0.22**	0.07	0.09	0.71	0.29	1.3
When I think about this course, I become anxious	14	**0.69**	**0.26**	–0.02	0.11	0.78	0.22	1.3
I am sure that I will do well on this course	16	**−0.66**	0.04	**0.44**	0.03	0.79	0.21	1.8
The contents and the assignments on this course make me unhappy	13	**0.64**	**0.32**	0.02	0.04	0.71	0.29	1.5
Studying the materials of this course feels hopeless	6	**0.61**	**0.34**	0.13	–0.01	0.62	0.38	1.7
In the beginning of this course I was confident that I will do well on this course	24	**−0.49**	0.14	0.18	0.04	0.27	0.73	1.5
I find the course exam a pleasant challenge	17	**−0.42**	**−0.21**	0.14	**0.21**	0.37	0.63	2.3
I am bored by this course	5	–0.14	**0.86**	0.00	0.01	0.64	0.36	1.1
The learning activities for this course are not interesting	18	–0.05	**0.85**	–0.09	0.02	0.74	0.26	1.0
I am fed up with the contents of this course	8	0.17	**0.79**	0.04	0.01	0.76	0.24	1.1
I find studying the course contents inspiring	1	–0.06	**−0.74**	0.11	0.15	0.65	0.35	1.1
Completing the assignments on this course irritates me	4	**0.37**	**0.54**	0.00	0.01	0.61	0.39	1.8
I enjoy learning new things on this course	15	–0.08	**−0.52**	**0.20**	0.04	0.43	0.57	1.4
I am extremely happy with the way I have invested my efforts in studying on this course	25	0.13	–0.06	**0.88**	–0.13	0.78	0.22	1.1
I feel ashamed not having prepared better for this course	10	–0.02	0.06	**−0.66**	**0.34**	0.63	0.37	1.5
I feel ashamed thinking I might have not prepared adequately for the exam in this course	12	0.07	–0.06	**−0.65**	**0.38**	0.66	0.34	1.7
I am extremely happy with my learning on this course	3	**−0.32**	–0.11	**0.56**	0.07	0.60	0.40	1.7
I am extremely happy with the way I have done with the homework in this course	7	–0.06	–0.01	**0.48**	–0.19	0.32	0.68	1.3
I feel optimistic about my preparation for the exam in this course	2	**−0.42**	0.02	**0.46**	0.06	0.50	0.50	2.0
Mistakes I make on the course (e.g., during the mathematical excercises) or when doing homework embarrass me	22	0.00	–0.01	–0.13	**0.80**	0.69	0.31	1.1
If I ask a question in class that others already certainly know the answer to, I feel embarrassed	21	0.18	**0.20**	–0.02	**0.64**	0.60	0.40	1.4
Contributing to discussions or asking questions on this course makes me anxious	20	0.04	0.01	0.04	**0.62**	0.40	0.60	1.0
I am embarrassed when I cannot complete the homework assignments on this course	19	**−0.21**	–0.03	0.02	**0.62**	0.37	0.63	1.2

	**Factor correlations**							
	**Anxiety and hopelessness**		**Boredom and anger**		**Effort-related emotions**		**Negative social emotions**	

Anxiety and hopelessness	1.00							
Boredom and anger	0.48		1.00					
Effort-related emotions	−0.36		−0.30		1.00			
Negative social emotions	0.22		0.10		−0.13		1.00	

*h^2^, communality; u^2^, uniqueness; com, complexity (the degree of cross-loadings).*

In the four-factor solution, the first factor comprised items related to anxiety and hopelessness together with items describing propositional attitudes related to learning on the course. The factor was named *anxiety and hopelessness*. Further, the items loading on this factor may also have reflected the student’s initial knowledge level concerning the subject matter: if the topics are unfamiliar, the student is unlikely to feel confident about performing well on the course (item 16) and is likely to feel unable of making it on the course (item 9). The Cronbach’s alpha for the scale was 0.90, and it would not have become higher with dropping any of the items. The average of the absolute values of primary factor loadings on this factor was 0.63, and two items had a communality value falling below 0.4. Together, these observations suggest that the convergent validity of this factor was moderate.

Items related to boredom and lack of interest had high loadings on the second factor, with negative loadings on items related to enjoying the course. The factor was thus dubbed *boredom and lack of interest*. A common denominator for the items might be the subjective difficulty of the course: if the student knows the course material well enough, the course may become boring, and if there are enough new and interesting things to learn, it will remain interesting (items 1 and 15). The Cronbach’s alpha for the scale was 0.88, and it would not have become higher with dropping any of the items. The average of the absolute values of primary factor loadings on this factor was 0.71, and the communalities of all items exceeded 0.4, suggesting that the convergent validity of the factor was satisfactory.

Items related to various positive and negative emotions loaded on the third factor. The common denominator for the items was the amount of effort expended on the course and the emotions resulting from this; consequently, the factor was dubbed *effort-related emotions*. The Cronbach’s alpha for the scale was 0.83, and it would not have become higher with dropping any of the items. The average of the absolute values of primary factor loadings on this factor was 0.62, and the communality of one of the items fell below 0.4, suggesting that the factor had moderate convergent validity. The fourth factor comprised items related to *negative emotions* (embarrassment, shame and anxiety) *in social situations* and was named correspondingly. The Cronbach’s alpha for the scale was 0.73, and it would have risen to 0.75 with dropping item e19. The average of primary factor loadings on this factor was 0.67, and one item had a communality value falling under 0.4. Together, these observations suggest that the convergent validity of the factor was moderate. The discriminant validity for the four-factor solution was satisfactory, as the interfactor correlations were no larger than 0.48 and most of them fell considerably under this value.

The results of the factor analysis differed somewhat from those obtained by Trigwell et al. (2012). The PCA solution of Trigwell et al. (2012) separated positive and negative emotions, while in our EFA, several items related to positive emotions had negative loadings on their respective factors. Further, while items related to anxiety and shame loaded on the same principal component in the analysis of Trigwell et al. (2012), in our analysis the feelings of shame loaded on the third (effort-related emotions) and fourth factor (*negative emotions in social situations*). Our factor *boredom and anger* was similar to the principal component *frustration* in Trigwell et al. (2012), with the exception that in our analysis interest-related emotions loaded negatively on this factor.

### Network Analysis

**FIGURE 3:**
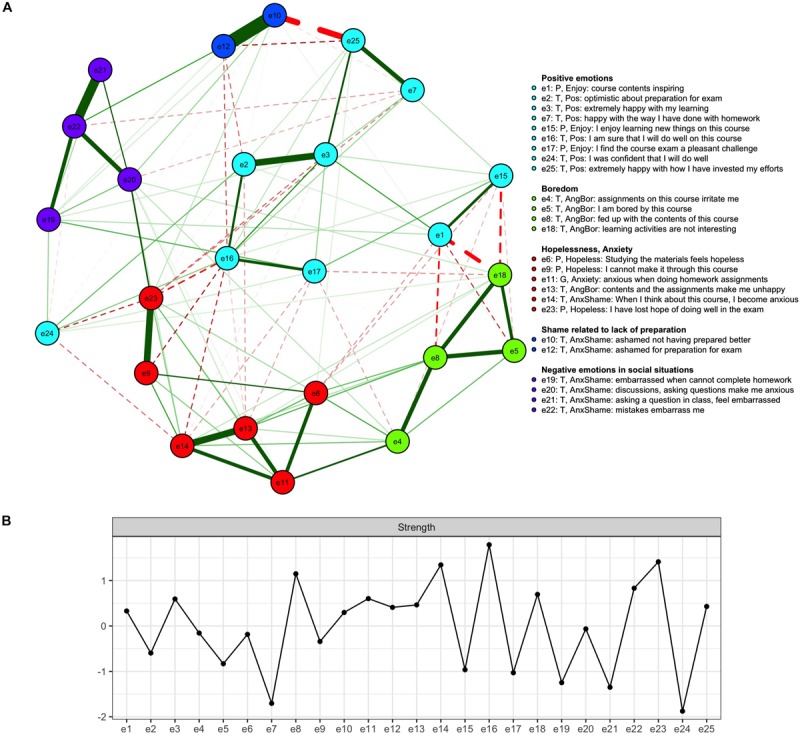
Network graph with the node groups selected based on the results of spinglass community detection algorithm (top, **A**) and the standardized strength centralities of all the nodes (bottom, **B**). The green solid lines represent positive connections among the nodes while the dashed red lines represent negative connections.

The network graph corresponding to the lasso-estimated partial correlations together with the standardized strength centralities for all the nodes are shown in Figure 3. The width and saturation of the line indicate the strength of the connection. In Figure 3A, the colors of the nodes were chosen based on the factor analysis reported above, whereas in Figure 3B they correspond to the results of the community detection analysis reported in Section “Community detection.”

When interpreting the network graphs, it may be instructive to think of what they would look like if a four-factor simple structure were the correct representation for this data. In that case, we would expect all items intended to measure a single factor to share edges with one another and to have only weak associations with the rest of the items. Sampling variation naturally makes things less clear-cut, even though lasso estimation is meant to address the issue.

In Figure 3, the negative emotions experienced in social situations come closest to exhibiting the pattern of partial correlations that we would expect if their interrelationships were indeed caused by an underlying latent variable. On the other hand, it is also possible that such associations arise because there is a central node that influences others in its immediate neighborhood. The factor analysis equivalent to such nodes would be an observed variable having a high loading on its respective factor. Among these four nodes, *being embarrassed by mistakes* (item 22) is the most strength central one. It also has the highest factor loading on the respective factor (Table 2).

Looking at the rest of the network graph in panel A, the similarly colored nodes do not form such clearly interconnected clusters. For instance, not all nodes related to *boredom and anger* share edges with one another. Rather, these nodes might be best interpreted as bearing family resemblance relations with each other, with strong connections between some of them and weak to non-existing ones between others. In this respect, it is of interest to note that the boredom-related nodes (e5, e8, and e18) share strong edges, while only one of them (e8) has a strong association with the anger-related node 4.

The interpretation of the lower part of the network graph in panel A, which is related to the emotions of anxiety and hopelessness, can also be based on the idea of family resemblance relationships among the items. The two nodes clearly related to anxiety (11 and 14) share a strong edge. On the other hand, their relationships with items 13 and 6 seem to be driven by two facts: all items have negative valence and mention the homework and/or contents of the course. The remaining strong edge obtains between nodes 23 and 9 (hopelessness), while both are only weakly connected with the remaining item expressing hopelessness (6). This may be because the former are related to a more general, overwhelming feeling and the latter to course material specifically.

Finally, when looking at the cyan items in Figure 3A, it is of interest to contrast the interpretation of the factor model with that of the network model. For instance, items 3, 7, and 25 loaded on the same factor (*effort*) and yet nodes 3 and 7 share only a weak edge in the graph. However, they both share a strong edge with node 25, which may actually function as a common cause for both: expending enough effort on the course may lead one to feel happy with one’s performance.

The relative importance of the nodes was quantified by strength centrality, shown in Figure 3B. The most central nodes included e16 (sure of doing well on the course), e23 (losing hope), e14 (anxiety), and e8 (Fed up with the course). Node 16 shared negative edges with nodes related to anxiety (e14), hopelessness (e9), (e23), shame (e12). Node 14 shared notable positive edges with unhappiness (e13) and another anxiety-related node (e11), and a negative edge with confidence (e24). Node 8 was negatively related to inspiration (e1), and shared a positive edge with irritation (e4), boredom (e5) and lack of interest (e18).

#### Network Accuracy and Stability

**FIGURE 4:**
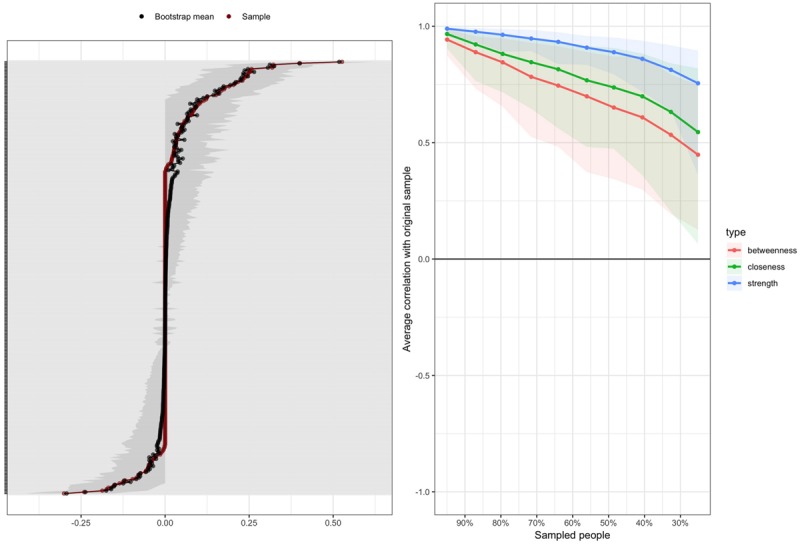
**(A)** Bootstrapped confidence intervals for all estimated edge weights. The red dots connected by the red line are the individual edge weights, and the gray lines the bootstrapped confidence intervals. Each individual gray line corresponds to one edge in the network. **(B)** Average correlations between the centrality indices calculated based on the whole sample and subsamples with fewer participants in the bootstrap analyses. The lines indicate the average correlations and the areas around them the range from the 2.5th to the 97.5th quintile.

The results of the edge-weight accuracy analysis are shown in Figure 4A. As the figure indicates, there is some overlap in the confidence intervals. This means that, on the one hand, obvious differences, such as those between negative and strongly positive edges, are readily interpretable. On the other hand, sampling variation affects the results such that, for instance, positive edges with small vs. medium weights in Figure 3 may in fact be equal on the population level.

The results of the sensitivity analysis are shown in Figure 4B. When looking at the betweenness and closeness centrality indices, it is seen that their correlations with the respective values in the full sample rapidly decreased when cases were dropped from the analysis; in other words, the values of these indices proved to be rather sensitive to the sample that was collected. The CS-index values were 0.13 for betweenness centrality, 0.205 for closeness centrality, and 0.594 for strength centrality. Consequently, only strength centrality is reported.

#### Community Detection

The global structure of the network was assessed using the spinglass community detection analysis. The most frequently obtained community structure, which the algorithm converged upon on 82.5% of the repetitions, is reported below. The next most frequent community structures occurred with probabilities of 7.4, 5.4, and 2.9%, with only few occurrences for other structures. In the most commonly obtained community structure, the items are grouped somewhat differently from the factor analysis, as can be seen by comparing Figure 3A with the results of the factor analysis. The most striking difference was observed concerning the positive emotions, which loaded on different factors but were grouped in the same community. Emotions related to anxiety and hopelessness formed another community, and those related to boredom a third one. The four items related to negative emotions in social situations formed a community of their own, with the remaining community consisting of two nodes related to shame arising due to a lack of effort. When looking at Figure 3A, it appears that node e24 is far removed from the other positive emotions. One should not, however, put too much weight on interpreting the visual appearance of the graph, as projecting the multidimensional data structure on a two-dimensional plane cannot represent all relationships between the nodes equally well. For this reason, we encourage the reader to repeat our analyses based on the R code and the data set provided at the osf.io page related to the present contribution. This enables the reader to examine the actual connection weights among the items and obtain further insights on the data.

## Discussion

Self-report instruments play an important role in quantitative studies of academic emotions, and the conclusions drawn in such studies depend on the structure of the instruments. In this contribution we have examined one such instrument using psychometric network models and latent factor models. The two kinds of models involve different conceptions of validity, lead to different practical conclusions, and are differently compatible with the theories underlying research into the nature of academic emotions (Scherer, 1984, 2009; Pekrun et al., 2002).

The central practical difference between network models and factor models is related to the idea of redundancy of items. Other things being equal, factor models encourage redundancy, since similar items will likely be highly correlated. Network models, on the other hand, discourage redundancy, as they involve the idea of *network components* as unique and causally autonomous parts of the network (Cramer et al., 2012). In what follows, we examine the structure of the presently introduced Finnish version of AEQ from these two points of view.

From the point of view of factor models, our first factor comprised items referring to emotions ranging from anxiety and hopelessness to general unhappiness with the course and its contents, i.e., various different emotions. Similarly, the second factor comprised items related to boredom, lack of interest and enjoyment (with negative loadings), while the common denominator for the items loading on the third factor was the effort (or the lack of it) expended on the course and the various emotions arising as a consequence. The fourth factor comprised different negative emotions (anxiety, embarrassment) related to social situations.

Such results pose a practical problem for the researcher committed to using reflective measurement models, which view the individual items as interchangeable indicators – or measurements – of the latent emotion. One option would be to drop the items that do not behave in the expected manner: for instance, we might be able to obtain a clearer factor related to only anxiety by dropping items related to hopelessness and general unhappiness. Similarly, factor analysis would suggest dropping items that cross-load on several factors, such as item 16 (*I am sure that I will do well on this course*). On the other hand, researchers must have a good scientific reason to initially include any given item in their instrument, which makes it a problematic practice to drop an item because it does not correlate enough with other items (low communality) or because it correlates too much with them (high cross-loadings) (Costello and Osborne, 2005).

Network models of academic emotions offer an interesting point of view to such questions. First, it can be noted that the meaning of item 16 comes close to how self-efficacy is defined: “people’s beliefs about their capabilities to produce designated levels of performance that exercise influence over events that affect their lives” (Bandura, 1994) and how generalized self-efficacy is measured (e.g., with an item “When I make plans, I am certain I can make them work” (Judge et al., 2002). Then, interpreted as a node in the network of emotions, it can be hypothesized that node e16 (*I am sure I will do well on this course*) inhibits feelings of hopelessness (e23, *having lost hope of doing well in the exam*; e9 *cannot make it through the course*) and anxiety (e14, *when I think about the course, I become anxious*). The interpretation serves as a causal hypothesis, as cross-sectional data does not allow testing causal assumptions. On the other hand, it is equally possible that the feelings of hopelessness and anxiety will inhibit experiences of self-efficacy.

Incidentally, one unique benefit of network analysis is that it enabled us to pinpoint the experiences that were central in the “emotional ecosystems” of the students. The concept of centrality differs from latent factor models, in which all observed variables are equal indicators of the latent variables. We assessed the centrality of the emotions using the strength index reported in Figure 3. Node e16 (*I am sure that I will do well on this course*) had the strongest connections with the other nodes. If the negative edges connecting the node to feelings of hopelessness and anxiety indeed signify causal relationships (something that cannot be tested or proved in the current study), strengthening the students’ feelings of self-efficacy would possibly reduce their feelings of hopelessness and anxiety. This interpretation is compatible with previous findings showing that self-efficacy is positively associated with experienced positive emotions and negatively associated with experiencing negative emotions while studying (Putwain et al., 2013; Roick and Ringeisen, 2017). Further, the network model leads to quite different practical conclusions than the factor model: while item 16 would possibly be removed from the questionnaire based on factor analyses due cross-loadings, it occupies a central position in a network model of academic emotions.

Further, the factor model and the network model lead to different conclusions concerning the overall structure of the instrument. In the network model, the issue was assessed using the spinglass community detection algorithm. While four factors were extracted, the spinglass algorithm converged most frequently on a five-community structure. Interestingly, positive emotions formed one community, while in the factor model they were aggregated together with the negative emotions with which they shared strong negative correlations. The result of the spinglass analysis accords with the human intuition that positive emotions belong together; simultaneously, it is congruent with the circumplex model of emotions (Lindquist, 2013). Let us continue by examining each of the communities more carefully.

In the community of nodes related to negative emotions in social situations, node e22 (*Being embarrassed by mistakes*) shared strong edges with the other nodes, which were not as strongly interconnected. The concepts of state and trait emotions may be helpful in interpreting this pattern of connections: e22 may be more trait-like than the other nodes. Nodes e19 (homework-related embarrassment) and e21 (embarrassment related to asking questions) could then be interpreted as concrete manifestations of the trait, whereas anxiety related to asking questions (e20) may be a causal effect of the trait: if the students know they will feel embarrassment when making a mistake, they may feel anticipatory anxiety in a situation where mistakes are likely.

The emotions of anger and boredom were clearly associated in our data similarly to the studies Govaerts and Grégoire (2008) and Trigwell et al. (2012), as the items related to these emotions loaded on the same factor also in our analysis. The network model, however, provided a novel point of view: perhaps this shared variation is best understood as family resemblance among the semantic contents of the items describing these emotions and perceptions. For instance, perceiving the learning activities as uninteresting (e18) was not directly related to getting irritated with the course assignments (e4) or feeling unhappy about the course contents and assignments (e13). Rather, e18 was only related to these through its association with e8, feeling fed up with the course contents, which is arguably semantically more similar to e4 than e18 is. On the other hand, the three boredom-related nodes (e5, e8, e18) were closely associated. The idea of family resemblance of the items’ semantic contents driving their associations may help to understand the apparent contradiction of an activating emotion (anger) and inhibiting emotion (boredom) loading on the same factor.

The pattern of results related to the emotions of anxiety and hopelessness can also be interpreted along similar lines. The two anxiety-related nodes (e11 and e14) shared a strong edge. On the other hand, their relationships with nodes e13 and e6 seemed to be driven by two facts: all items mentioned homework or course contents and involved an emotion with a negative valence. The hopelessness-related nodes e23 and e9 similarly shared a strong edge, even though both were only weakly connected to the remaining hopelessness-related node (e6). This may be because the former are related to a general, overwhelming feeling and the latter to course material specifically, driving its correlation with item 11, as noted above.

Finally, it is of interest that all positive emotions were grouped in the same community. They shared positive edges with one another, and negative ones with nodes related to negative emotions, which is in keeping with the circumplex model of emotions. Further, it is of interest to compare the results of the network analysis with those of the factor analysis when it comes to positive emotions. In the factor model, items 3 and 7 (*happy with own learning*) and 25 (*happy with own effort*) loaded on the same factor (effort) even though nodes e3 and e7 shared only a weak edge in the network graph. However, both shared a strong edge with 25, which may have functioned as a common cause for both: expending enough effort on the course may have caused the students to feel happy with their performance and learning. Importantly, such hypothetical causal relationships would not be revealed based on factor analysis only. Again, from the practical point of view, item e2 (*optimistic about preparation for exam*), shared positive edges with other positive emotions and negative edges with negative emotions such as e12 (*ashamed for preparation for exam*) and e13 (*unhappy about course contents and assignments*). In the factor model, item e2 loaded equally strongly on two factors and would likely have been removed based on factor analytic considerations. However, when viewed as a part of the network model of academic emotions, this pattern of associations seems natural: why would not feelings of optimism be negatively related to feelings of unhappiness, for instance?

As seen above, the edges of the network models can be given different interpretations: they may be reflect hypothetical causal connections, the semantic similarities of the contents of the questionnaire items, or be due to the effects of unmodeled latent variables. Constructing both a network model and a factor model allows the researcher to formulate educated guesses on which of the associations are due to latent variables and which ones are potentially causal in nature. In a factor model it is assumed that all the connections are due to latent variables, whereas in a network model it is assumed that none of them are. We believe the correct answer lies somewhere between these two extremes, as do others: for an extensive discussion of the topic, see Fried and Cramer (2016). Recent research has in fact resulted in psychometric models that enable combining latent factors as a part of a network model (Epskamp et al., 2017). Such models might provide a useful also in the present context: the above-mentioned semantically similar nodes (e.g., the boredom-related nodes e5, e8, and e18) could be represented by a latent factor, which would then function as a single node in the network model.

Additional practical considerations may also favor one modeling strategy over the other. Negative correlations among items, represented either as negative factor loadings or negative edges, are a case at point. In factor models, items with a negative loading may pose a problem for naming the factor. In this situation it is tempting to reverse the content of the items with negative loading to give the latent variable a congruent interpretation. This is problematic, however, as coming up with the opposite of a verbal statement is to a degree a subjective decision. Further, it is an empirical question whether the correlation of the opposite verbal statement is the opposite number of the correlation in question. For instance, there is a strong negative correlation between items 15 (I enjoy learning new things on the course) and 18 (learning activities are not interesting) and in our factor model, they load on the same factor with opposite signs. But after coming up with the verbal opposite of item 15, perhaps as *I need to force myself to study the course contents*, the relationship of this item and item 18 becomes a novel empirical question and should be investigated separately. On the other hand, when data analysis is based on a network model, the relationship can be interpreted as enjoyment of learning inhibiting feelings of anger or boredom (or vice versa). Due to these practical considerations, we prefer the results of the community detection analysis as a representation of the global correlational structure of the questionnaire in this study.

### Theoretical Limitations of the Present Study

Whether we consider academic emotions as emergent phenomena or natural kinds, we must ask which emotions to differentiate from one another, and consequently, which items to include in a questionnaire. Both the questionnaire used presently and the AEQ include items related to embarrassment among the items intended to measure shame, which presupposes that embarrassment and shame are parts of the same emotion, perhaps only differing with regards to the intensity of the affect. Empirical research, however, shows this not to be the case (Keltner, 1996; Tangney et al., 1996) at least in the context of American university students.

Shame and embarrassment have been found to be qualitatively, not merely quantitatively, different emotions: they are both associated with specific kinds of precipitating factors, consequences, and the contexts in which they may be felt. Shame is an emotion signaling a moral transgression, unlike embarrassment, which is related to (minor) social transgressions. Shame may be a private emotion in contrast to embarrassment: while embarrassment may result in laughter and jokes, shame is more likely to lead to self-directed anger and apologies. Embarrassment is also more likely to involve physiological reactions such as blushing or increased heart rate than shame. Embarrassment is felt when the actor has no responsibility for the consequences and when others are likely to be amused by what happened – unlike shame (Keltner, 1996; Tangney et al., 1996). Moreover, at least in the American context, shame has been found to be very rarely the consequence of cognitive shortcomings, unlike embarrassment (Keltner, 1996). It may thus be reasonable to aim at separating the emotions of shame and embarrassment in the questionnaire.

Furthermore, it is not self-evident that the complex emotions of pride, hope, anxiety, shame and hopelessness are similarly structured across cultures, raising the question of whether these differences were adequately taken into account when developing the Finnish translation of the questionnaire. There are notable differences across cultures in how emotions are expressed and displayed (Mesquita and Frijda, 1992), and even though Govaerts and Grégoire (2008) included an item related to expressing anger by throwing books out of the window, such behavior would have been uncommonly impulsive in the Finnish context.

### Methodological Limitations of the Present Study

As the present data is not based on the use of a questionnaire specifically developed to investigating academic emotions as networks of interrelated phenomena, we are only able to present a general outline of how academic emotions could be investigated using network models. We hope that future studies will see questionnaire instruments specifically developed from this starting point.

Further, we were not able to interpret the betweenness and closeness centrality indices, as their estimates did not prove stable in the present data. One reason for this may be the smallish size of the present data, and it might prove fruitful to base future studies on larger sample sizes. Similarly, the nature of the sample may affect the results that were obtained: it is possible that the factor or network structures of academic emotions vary between disciplines. The present results, being based on the experiences of students of technology and engineering, may thus not apply to, say, students of non-professional subjects. In addition, the present sample consisted of mostly male students, and it may be that male and female students may experience different kinds of emotions during a course of a technical nature.

In addition, the estimation method used in producing the network models, the graphical lasso, is suitable if a sparse network model (few edges between the nodes) is the correct representation of the data. Applying the lasso will always result in a sparse network, whether in reality a sparse network is a good description of the phenomenon or not (Epskamp et al., 2017). Our result is thus sensible only insofar as it is reasonable to suspect that many of the nodes in our network models should indeed not be connected to each other.

Further, the centrality indices may reflect the presence of unmodeled latent variables, which may inflate their values. For instance, the extremely strong connection between nodes 10 and 12, both related to shame arising from lack of preparation, may actually reflect the presence of this latent variable. If that is the case, it might have been a good idea to leave one of these variables out of the network model in order not to artificially inflate the centrality estimates of these two variables (Fried and Cramer, 2016).

Naturally, our methodological choices related to the factor analysis can also be problematized. We performed the factor analysis based on polychoric correlations, and the calculation method used in the present study assumes the normality of the underlying continuous variables. The results might have been affected by calculating the polychoric correlations based on the copula method (Ekström, 2011). This, of course, applies equally to the network analyses, and such questions remain to be addressed in the future in more methodologically oriented studies. Further, the minres rotation method has been shown to be sensitive to the presence of so-called local solutions (Hattori et al., 2017), even though it performs well with complex loading structures. Because of this, it cannot be ruled out that the present results of the factor analysis would be specific to the sample at hand, especially due to the smallish sample size.

### Conclusion and Ideas for Further Studies

Considerations such as those expressed in the previous sections naturally lead one to think about the response format of the AEQs that are currently in use. They associate a given emotion with a given likely precipitating factor, even though other emotions could likely be felt under the same circumstances. For example, in the questionnaire used in the present study, there is an item that states “Contributing to discussions or asking questions on this course makes me anxious,” but no item related to feeling happy or enthusiastic about these things. Instead of formulating items that conflate the presence of a precipitating factor (here: asking a question or taking part in a conversation) and experiencing a certain emotion (here: anxiety), another possible response format would be to ask the respondents to report the intensity of any emotion they may have felt in conjunction with the precipitating factor. Concretely, that would entail writing items such as:

When taking part in a conversation in which the whole class participates, I feel the following:

Anxiety
Annoyance
Boredom
Joy
[…]

with each emotion being associated with its own Likert response scale, and perhaps each emotion being reflected by a set of words (e.g., anger by “anger,” “mad,” “rage,” and “pissed off” – words assumedly used by the participants themselves to refer to these emotions). A format similar to this has been used in the Discrete Emotions Questionnaire (Harmon-Jones et al., 2016). This format would have the added benefit of not being sensitive to overlapping non-emotional content that may have caused some of the associations in the questionnaire used in the present study as pointed out above. In addition to the format of the questionnaire, the introduction of various reference measures would be of interest in future studies. For instance, how do the various components of academic emotions relate to physiological measures?

In summary, we have suggested a network perspective to modeling academic emotions, which we believe to be useful for the field as a whole. In particular, the control value theory of academic emotions (Pekrun et al., 2007) seems to us compatible with the network perspective, which enables modeling emotions as interacting networks of affective, cognitive, motivational and physiological components. In addition, the cross-sectional between-persons network models can be interpreted as aggregations of person-specific models (Cramer et al., 2012). This thought motivates interesting possibilities for future research, as the network representations of emotions may differ across individuals: for one student, boredom and anger may occur together and be subsumed under the common rubric of frustration, while for another, boredom and anger may be distinct experiences. Longitudinal data is naturally needed for examining such questions related to intra-individual variation.

Network analysis has only recently been applied as a psychometric method, and the novelty of the method means that methodological and philosophical open questions remain. One important methodological question that needs to receive more attention in future studies concerns the most suitable method of carrying out community detection analysis within network psychometrics. Methodologically oriented future studies will surely shed light on the issue and provide applied researchers with a set of recommendations on best practices. Further, intriguing philosophical questions remain to be solved. For instance: can latent variables as mereological sums be thought of as emergent properties either in the ontological or the epistemological sense (O’Connor and Wong, 2015), i.e., do the latent variables thus conceived have properties that are not shared by the observed variables? If they do, questions related to whole-part causation (top–down causation) arise, whereas it is characteristic for the network psychometrics literature as it currently stands to think of wholes composed of parts from the bottom up (for example when thinking of depression as a combination of symptoms (Fried, 2015). It is illustrative to consider that the same mental state, such as feeling jealousy, may have multiple realizations as networks of interrelated variables. For instance, jealousy in one person may involve anger and violent intentions as component parts, whereas in someone else they may be replaced by intense sorrow; yet we speak of the same emotion. Perhaps, then, general psychological properties are determined by both local interactions among components parts (from the bottom up) and the global influence of the person as the context in which they occur (top–down influence).

All in all, intriguing substantive, methodological and philosophical questions remain to be investigated within the arising field of network psychometrics, and in the domain of emotion research in particular. All analysis code and the data that our contribution is based on are published on the web page for the pre-print of this article^1^ for the benefit of researchers interested in applying network analysis in their own work.

## Data Availability Statement

All datasets generated for this study are included in the article/Supplementary Material.

## Ethics Statement

Ethical review and approval was not required for the study on human participants in accordance with the local legislation and institutional requirements. The patients/participants provided their written informed consent to participate in this study.

## Author Contributions

MM, TH, and AP designed the study. TH and AP designed the questionnaire, collected the data, and wrote sections detailing the sample of students and data collection. MM performed the data analyses and wrote the first draft of the manuscript. All authors approved the final manuscript.

## Conflict of Interest

The authors declare that the research was conducted in the absence of any commercial or financial relationships that could be construed as a potential conflict of interest.

## Footnotes

1 https://osf.io/mnbst/

## References

[B1] Asikainen H. Hailikari T. Mattson M. (2017). The interplay between academic emotions, psychological flexibility and self-regulation as predictors of academic achievement. *J. Further and Higher Education* 42 439–453. 10.1080/0309877x.2017.1281889

[B2] Bandura A. (1994). “Self-efficacy,” in *Encyclopedia of Human Behavior*, Vol. 4, ed. Ramachaudran V. S. (New York: Academic Press), 71–81.

[B3] Bollen K. A. Bauldry S. (2011). Three Cs in measurement models: causal indicators, composite indicators, and covariates. *Psychol. Methods* 16, 265–284. 10.1037/a0024448 21767021 PMC3889475

[B4] Borsboom D. (2017). A network theory of mental disorders. *World Psychiatry* 16 5–13. 10.1002/wps.20375 28127906 PMC5269502

[B5] Borsboom D. Cramer A. O. (2013). Network analysis: An integrative approach to the structure of psychopathology. *Annual Review of Clinical Psychology* 9 91–121. 10.1146/annurev-clinpsy-050212-185608 23537483

[B6] Borsboom D. Cramer A. O. Kievit R. A. Scholten A. Z. Franiæ S. (2009). “The end of construct validity,” in *The Concept of Validity: Revisions, New Directions and Applications, Oct, 2008*, ed. Lissitz R. W. (Charlotte, NC: IAP Information Age Publishing), 135–170.

[B7] Borsboom D. Mellenbergh G. J. Van Heerden J. (2003). The theoretical status of latent variables. *Psychological Review* 110 203–219. 10.1037/0033-295X.110.2.203 12747522

[B8] Briggs N. E. MacCallum R. C. (2003). Recovery of weak common factors by maximum likelihood and ordinary least squares estimation. *Multivariate Behavioral Research* 38 25–56. 10.1207/S15327906MBR3801_2 26771123

[B9] Christensen A. P. Golino H. Silvia P. (2019). A psychometric network perspective on the measurement and assessment of personality traits. *PsyArXiv* 10.31234/osf.io/ktejp

[B10] Costantini G. Epskamp S. Borsboom D. Perugini M. Mõttus R. Waldorp L. J. (2015). State of the aRt personality research: A tutorial on network analysis of personality data in R. *Journal of Research in Personality* 54 13–29. 10.1016/j.jrp.2014.07.003

[B11] Costello A. B. Osborne J. (2005). Best practices in exploratory factor analysis: Four recommendations for getting the most from your analysis. *Practical Assessment, Research, and Evaluation* 10 7.

[B12] Cramer A. O. Sluis S. Noordhof A. Wichers M. Geschwind N. Aggen S. H. (2012). Measurable like temperature or mereological like flocking? on the nature of personality traits. *European Journal of Personality* 26 451–459. 10.1002/per.1879

[B13] Cramer A. O. Van der Sluis S. Noordhof A. Wichers M. Geschwind N. Aggen S. H. (2012). Dimensions of normal personality as networks in search of equilibrium: You can’t like parties if you don’t like people. *European Journal of Personality* 26 414–431. 10.1002/per.1866

[B14] Cronbach L. J. Meehl P. E. (1955). Construct validity in psychological tests. *Psychological Bulletin* 52 281 10.1037/h0040957 13245896

[B15] Csardi G. Nepusz T. (2006). The igraph software package for complex network research. *InterJournal, Complex Systems* 1695 1–9.

[B16] Edwards J. R. Bagozzi R. P. (2000). On the nature and direction of relationships between constructs and measures. *Psychological Methods* 5 155–174. 10.1037/1082-989x.5.2.155 10937327

[B17] Ekström J. (2011). *A generalized definition of the polychoric correlation coefficient. Unpublished manuscript.* University of California, Los Angeles: Department of Statistics.

[B18] Epskamp S. Borsboom D. Fried I. (2017). Estimating psychological networks and their accuracy: A tutorial paper. *Behavior Research Methods* 50 195–212. 10.3758/s13428-017-0862-1 PMC5809547 28342071

[B19] Epskamp S. Cramer A. O. Waldorp L. J. Schmittmann V. D. Borsboom D. (2012). Qgraph: Network visualizations of relationships in psychometric data. *Journal of Statistical Software* 48 1–18.

[B20] Epskamp S. Fried I. (2016). A tutorial on regularized partial correlation networks. *ArXiv* 10.1037/met0000167 29595293

[B21] Epskamp S. Kruis J. Marsman M. (2017). Estimating psychopathological networks: Be careful what you wish for. *PloS One* 12:e0179891 10.1371/journal.pone.0179891 PMC5482475 28644856

[B22] Epskamp S. Maris G. Waldorp L. J. Borsboom D. (2018). “Network psychometrics,” in *The wiley handbook of psychometric testing: A multidisciplinary reference on survey, scale and test development*, eds Irwing P. Hughes D. Booth T. (New York: John Wiley & Sons), 10.1002/9781118489772.ch30

[B23] Epskamp S. Rhemtulla M. Borsboom D. (2017). Generalized network psychometrics: Combining network and latent variable models. *Psychometrika* 82 904–927. 10.1007/s11336-017-9557-x 28290111

[B24] Fortunato S. (2010). Community detection in graphs. *Physics Reports* 486 75–174. 10.1016/j.physrep.2009.11.002

[B25] Fortunato S. Castellano C. (2007). Community structure in graphs. *arXiv* arXiv:0712.2716,

[B26] Fortunato S. Hric D. (2016). Community detection in networks: A user guide. *Physics Reports* 659 1–44. 10.1016/j.physrep.2016.09.002

[B27] Fried E. I Cramer A. O. (2016). Moving forward: Challenges and directions for psychopathological network theory and methodology. *Perspect Psychol Sci* 12 999–1020. 10.1177/1745691617705892 28873325

[B28] Fried E. I. (2015). Problematic assumptions have slowed down depression research: Why symptoms, not syndromes are the way forward. *Frontiers in Psychology* 6:309. 10.3389/fpsyg.2015.00309 25852621 PMC4369644

[B29] Friedman J. Hastie T. Tibshirani R. (2008). Sparse inverse covariance estimation with the graphical lasso. *Biostatistics* 9 432–441. 10.1093/biostatistics/kxm045 18079126 PMC3019769

[B30] Fruchterman T. Reingold E. (1991). Graph drawing by force−directed placement. *Software: Practice and Experience* 21 1129–1164. 10.1002/spe.4380211102

[B31] Govaerts S. Grégoire J. (2008). Development and construct validation of an academic emotions scale. *International Journal of Testing* 8 34–54. 10.1080/15305050701808649

[B32] Harman H. H. Jones W. H. (1966). Factor analysis by minimizing residuals (minres). *Psychometrika* 31 351–368. 10.1007/bf02289468 5221131

[B33] Harmon-Jones C. Bastian B. Harmon-Jones E. (2016). The discrete emotions questionnaire: A new tool for measuring state self-reported emotions. *PloS One* 11:e0159915. 10.1371/journal.pone.0159915 27500829 PMC4976910

[B34] Hattori M. Zhang G. Preacher K. J. (2017). Multiple local solutions and geomin rotation. *Multivariate behavioral research* 52 720–731. 10.1080/00273171.2017.1361312 28952786

[B35] Judge T. A. Erez A. Bono J. E. Thoresen C. J. (2002). Are measures of self-esteem, neuroticism, locus of control, and generalized self-efficacy indicators of a common core construct? *Journal of Personality and Social Psychology* 83 693–710. 10.1037/0022-3514.83.3.693 12219863

[B36] Kane M. T. (2006). Validation. *Educational Measurement* 4 17–64.

[B37] Keltner D. (1996). Evidence for the distinctness of embarrassment, shame, and guilt: A study of recalled antecedents and facial expressions of emotion. *Cognition & Emotion* 10 155–172. 10.1080/026999396380312

[B38] Ketonen E. Lonka K. (2012). Do situational academic emotions predict academic outcomes in a lecture course? *Procedia-Social and Behavioral Sciences* 69 1901–1910. 10.1016/j.sbspro.2012.12.144

[B39] Kirkpatrick S. Gelatt C. D. Jr. Vecchi M. P. (1983). Optimization by simulated annealing. *Science (New York, N.Y.)* 220 671–680. 10.1126/science.220.4598.671 17813860

[B40] Kline R. B. (2011). *Principles and practice of structural equation modeling* (, Third Edn New York: The Guilford Press.

[B41] Knekta E. Runyon C. Eddy S. (2019). One size doesn’t fit all: Using factor analysis to gather validity evidence when using surveys in your research. *CBE—Life Sciences Education* 18 rm1 10.1187/cbe.18-04-0064 PMC6757227 30821600

[B42] Korhonen V. Mattsson M. Inkinen M. Toom A. (2019). Understanding the multidimensional nature of student engagement during the first year of higher education. *Frontiers in Psychology* 10:1056. 10.3389/fpsyg.2019.01056 PMC6524002 31133948

[B43] Kossakowski J. Epskamp S. Kieffer J. van Borkulo C. Rhemtulla M. Borsboom D. (2016). The application of a network approach to health-related quality of life (HRQoL): Introducing a new method for assessing HRQoL in healthy adults and cancer patients. *Quality of Life Research* 25 781–792. 10.1007/s11136-015-1127-z 26370099 PMC4830856

[B44] Lindquist K. A. (2013). Emotions emerge from more basic psychological ingredients: A modern psychological constructionist model. *Emotion Review* 5 356–368. 10.1177/1754073913489750

[B45] Markus K. A. (2014). Unfinished business in clarifying causal measurement: Commentary on bainter and bollen. *Measurement: Interdisciplinary Research & Perspectives* 12 146–150. 10.1080/15366367.2014.980106

[B46] Mattsson M. T. (2019). Network models of driver behavior. *PeerJ* 6 e6119. 10.7717/peerj.6119 30647993 PMC6330205

[B47] Mesquita B. Frijda N. H. (1992). Cultural variations in emotions: A review. *Psychological Bulletin* 112 179–204. 10.1037/0033-2909.112.2.179 1454891

[B48] Messick S. (1987). Validity. *ETS Research Report Series* 1987 i–208.

[B49] Molenaar P. C. (2010). Latent variable models are network models. *Behavioral and Brain Sciences* 33 166–166. 10.1017/S0140525X10000798 20584385

[B50] National Advisory Board on Research Ethics (2009). *Ethical principles of research in the humanities and social and behavioural sciences and proposals for ethical review.* Available online at: http://www.tenk.fi/sites/tenk.fi/files/ethicalprinciples.pdf (accessed December 26, 2019)

[B51] O’Connor T. Wong H. Y. (2015). “Emergent properties,” in *The stanford encyclopedia of philosophy* (Summer 2015 edition), ed. Zalta E. N. (Stanford, CA: Stanford University).

[B52] Opsahl T. Agneessens F. Skvoretz J. (2010). Node centrality in weighted networks: Generalizing degree and shortest paths. *Social Networks* 32 245–251. 10.1016/j.socnet.2010.03.006

[B53] Pekrun R. (2006). The control-value theory of achievement emotions: Assumptions, corollaries, and implications for educational research and practice. *Educational Psychology Review* 18 315–341. 10.3109/0142159X.2012.643265 22364457

[B54] Pekrun R. Linnenbrink-Garcia L. (2012). “Academic emotions and student engagement,” in *Handbook of Research on Student Engagement*, eds Christenson S. Reschly A. Wylie C. (Boston, MA: Springer), 259–282. 10.1007/978-1-4614-2018-7_12

[B55] Pekrun R. Frenzel A. C. Goetz T. Perry R. P. (2007). “The control-value theory of achievement emotions: An integrative approach to emotions in education,” in *Educational psychology series. Emotion in education*, eds Schutz P. A. Pekrun R. (Cambridge, MA: Elsevier Academic Press), 13–36. 10.1016/b978-012372545-5/50003-4

[B56] Pekrun R. Goetz T. Perry R. P. (2005). *Achievement Emotions Questionnaire (AEQ). User’s manual*. Munich: University of Munich, 14.

[B57] Pekrun R. Goetz T. Frenzel A. C. Barchfeld P. Perry R. P. (2011). Measuring emotions in students’ learning and performance: The achievement emotions questionnaire (AEQ). *Contemporary Educational Psychology* 36 36–48. 10.1016/j.cedpsych.2010.10.002

[B58] Pekrun R. Goetz T. Titz W. Perry R. P. (2002). Academic emotions in students’ self-regulated learning and achievement: A program of qualitative and quantitative research. *Educational Psychologist* 37 91–105. 10.1207/s15326985ep3702_4

[B59] Putwain D. Sander P. Larkin D. (2013). Academic self-efficacy in study-related skills and behaviours: Relations with learning−related emotions and academic success. *British Journal of Educational Psychology* 83 633–650. 10.1111/j.2044-8279.2012.02084.x 24175686

[B60] R Core Team. (2016). *R: A language and environment for statistical computing [computer software].* Vienna, Austria: R Foundation for Statistical Computing.

[B61] Reichardt J. Bornholdt S. (2006). Statistical mechanics of community detection. *Physical Review E* 74 016110. 16907154 10.1103/PhysRevE.74.016110

[B62] Revelle W. (2014). *Psych: Procedures for personality and psychological research [computer software].* Evanston: Northwestern University.

[B63] Roick J. Ringeisen T. (2017). Self-efficacy, test anxiety, and academic success: A longitudinal validation. *International Journal of Educational Research* 83 84–93. 10.1016/j.ijer.2016.12.006

[B64] Schafer J. L. Graham J. W. (2002). Missing data: Our view of the state of the art. *Psychological Methods* 7 147. 10.1037/1082-989x.7.2.147 12090408

[B65] Scherer K. R. (1984). On the nature and function of emotion: A component process approach. *Approaches to Emotion* 2293 31. 18363269

[B66] Scherer K. R. (2009). Emotions are emergent processes: They require a dynamic computational architecture. *Philosophical Transactions of the Royal Society B: Biological Sciences* 364 3459–3474. 10.1098/rstb.2009.0141 19884141 PMC2781886

[B67] Schmitt T. A. Sass D. A. (2011). Rotation criteria and hypothesis testing for exploratory factor analysis: Implications for factor pattern loadings and interfactor correlations. *Educational and Psychological Measurement* 71 95–113. 10.1177/0013164410387348

[B68] Tangney J. P. Miller R. S. Flicker L. Barlow D. H. (1996). Are shame, guilt, and embarrassment distinct emotions? *Journal of Personality and Social Psychology* 70 1256. 10.1037/0022-3514.70.6.1256 8667166

[B69] Trigwell K. Ellis R. A. Han F. (2012). Relations between students’ approaches to learning, experienced emotions and outcomes of learning. *Studies in Higher Education* 37 811–824. 10.1080/03075079.2010.549220

[B70] van Bork R. Rhemtulla M. Waldorp L. J. Kruis J. Rezvanifar S. Borsboom D. (2019). Latent variable models and networks: Statistical equivalence and testability. *Multivariate Behavioral Research* 1–24. 10.1080/00273171.2019.1672515 31617420

[B71] Yang Z. Algesheimer R. Tessone C. J. (2016). A comparative analysis of community detection algorithms on artificial networks. *Scientific Reports* 6 30750. 10.1038/srep30750 27476470 PMC4967864

[B72] Zeidner M. (1998). *Test anxiety: The state of the art.* Berlin: Springer Science & Business Media.

